# Supplementary material for: OX40 ligand newly expressed on bronchiolar progenitors mediates influenza infection and further exacerbates pneumonia
Source: EMBO Mol Med. 2016 Mar 14;8(4):422–36. doi: 10.15252/emmm.201506154 (PMC4818750; doi:10.15252/emmm.201506154)
Supplement: Supplementary file 1 — Appendix [file EMMM-8-422-s001.pdf]

Appendix PDF

Bronchiolar progenitors paradoxically exacerbate influenza pneumonia via OX40 ligand

Taizou Hirano, Toshiaki Kikuchi, Naoki Tode, Arif Santoso, Mitsuhiro Yamada, Yoshiya Mitsuhashi, Riyo Komatsu, Takeshi Kawabe, Takeshi Tanimoto, Naoto Ishii, Yuetsu Tanaka, Hidekazu Nishimura, Toshihiro Nukiwa, Akira Watanabe, and Masakazu Ichinose

Contents

Page 1. **Appendix Figure S1** and the legend.

Page 2. **Appendix Figure S2** and the legend.

Page 3. **Appendix Figure S3** and the legend.

Page 4. **Appendix Figure S4** and the legend.

Page 5. **Appendix Figure S5** and the legend.

Page 6. **Appendix Figure S6** and the legend.

Page 7. **Appendix Figure S7** and the legend.

Page 8. **Appendix Figure S8** and the legend.

Page 9. **Appendix Figure S9** and the legend.

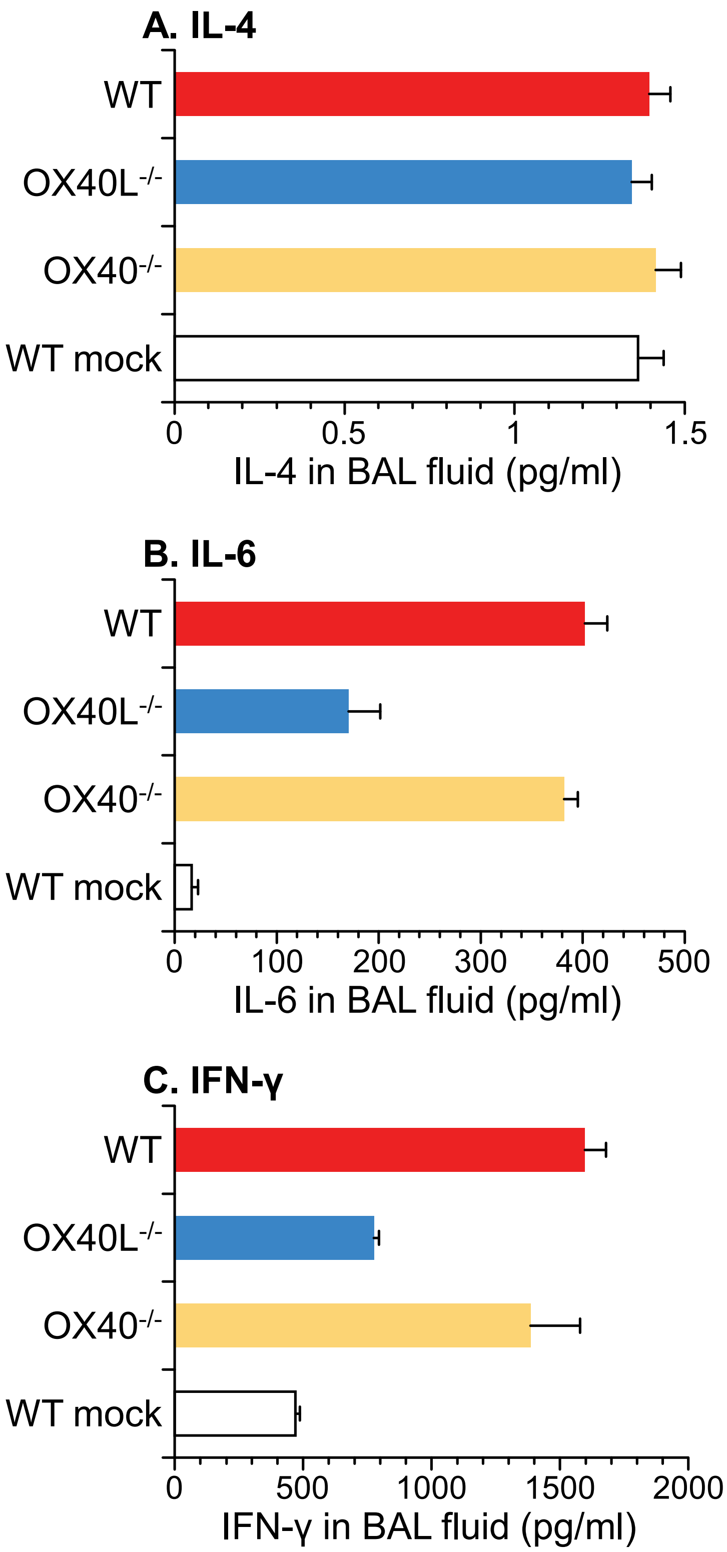

Appendix Figure S1. Cytokine levels in bronchoalveolar lavage (BAL) fluids of mice after influenza infection. Wild-type (WT), OX40L-deficient ($OX40L^{-/-}$), and OX40-deficient ($OX40^{-/-}$) mice were intratracheally infected with a lethal dose of influenza A/H1N1 virus (PR8 strain). Controls included wild-type mice treated with saline (mock). Seven days after the infection, BAL fluids were obtained from the mice to determine the concentrations of cytokines, such as interleukin (IL)-4, IL-6, and interferon (IFN)-γ, by using ELISA kits. Data are shown as the mean ± standard error ($n = 4$).

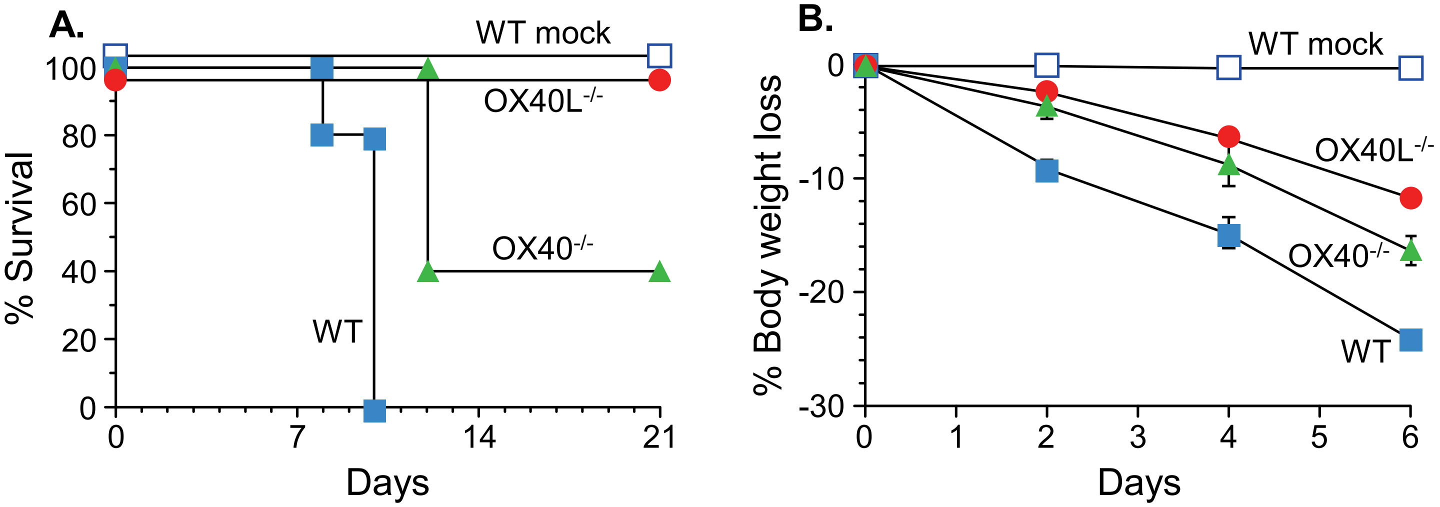

Appendix Figure S2. Influenza A/H3N2 was used for the infection to mice lacking OX40L and OX40. This study was similar to that in **Fig. 1A**, but mice were infected with a lethal dose of influenza A/H3N2 virus. Controls included wild-type mice treated with saline (mock). The susceptibility was determined by the survival of the mice (*n* =5, **A**), and body weight change (*n* =5, **B**). For panel **B**, data are shown as the mean ± standard error.

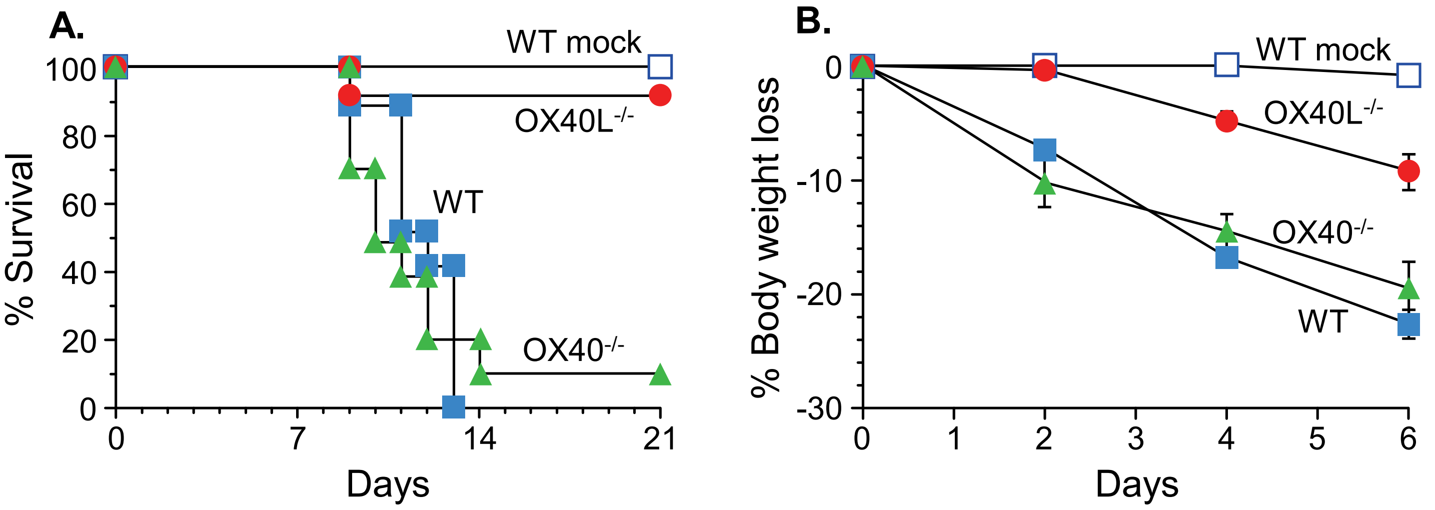

Appendix Figure S3. Another lethal dose of influenza A/H1N1 was used for the infection to mice lacking OX40L and OX40. This study was similar to that in **Fig. 1A**, but mice were infected with another lethal dose of influenza A/H1N1 virus (PR8 strain, 2.5 times the minimal lethal dose). Controls included wild-type mice treated with saline (mock). The susceptibility was determined by the survival of the mice (n = 10, **A**), and body weight change (n = 3, **B**). For panel **B**, data are shown as the mean ± standard error.

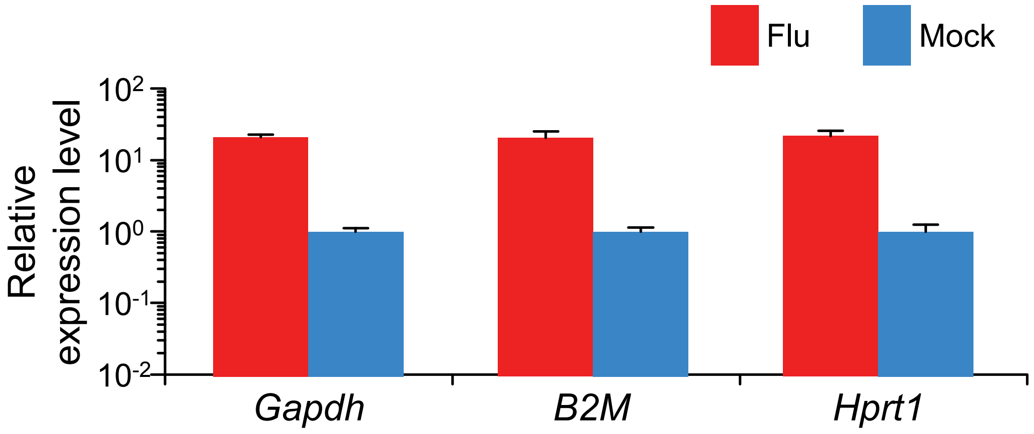

Appendix Figure S4. *OX40L* gene expression in bronchiolar progenitors. Wild-type mice were intratracheally infected with a lethal dose of influenza A/H1N1 virus (Flu) or saline (Mock), and 7 days later their bronchiolar progenitors were evaluated. By quantitative RT-PCR, the gene expression levels were normalized to the mouse *Gapdh*, *B2M* (beta-2-microglobulin), or *Hprt1* (hypoxanthine phosphoribosyl-transferase 1). The normalized expression levels were analyzed relative to the bronchiolar progenitors of mock-infected mice. Data are presented as the mean ± standard error of $n = 3$ per group.

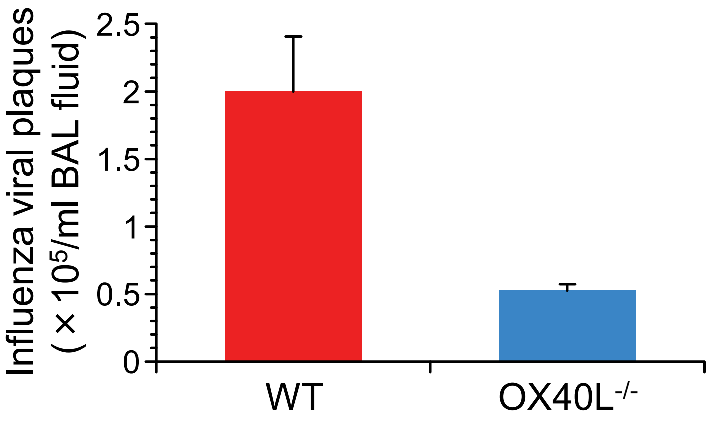

Appendix Figure S5. The deficiency of OX40L lowers the viral replication in the lung. Wild-type (WT) and OX40L$^{-/-}$ mice were intratracheally infected with a lethal dose of influenza A/H1N1 virus. Seven days later the viral burden in the lung tissue was determined by the number of the influenza plaques in the bronchoalveolar lavage (BAL) fluid. Data are shown as the mean ± standard error (*n* = 4).

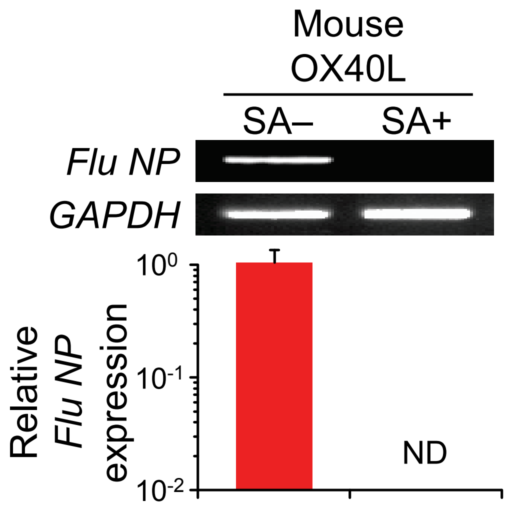

Appendix Figure S6. Sialidase treatment prevents the influenza infection mediated by mouse OX40L. Mouse OX40L-transfected MDCK cells were pretreated with or without sialidase (SA) for 24 hours. After the sialidase treatment, the cells were infected in vitro with influenza A/H1N1 virus, and 24 hours later the levels of influenza virus *NP* gene expression were analyzed by semi-quantitative and quantitative RT-PCR. The intensity was quantified relative to sialidase-untreated cells. Endogenous canine *GAPDH* mRNA expression was used as a control. Data are shown as the mean ± standard error (n = 3). ND, not detectable.

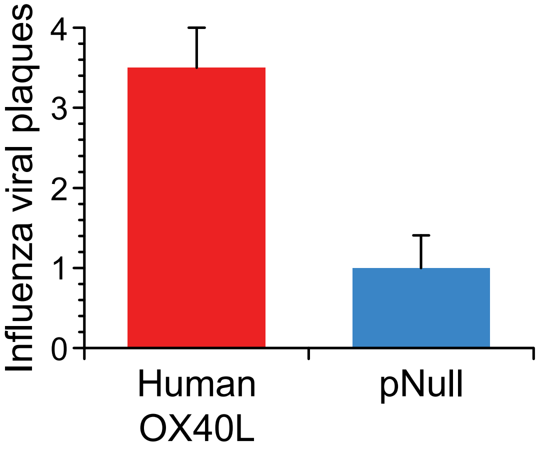

Appendix Figure S7. Human OX40L mediates influenza infection in vitro. Human OX40L-transfected MDCK cells in a 12-well plate were infected in vitro with influenza A/H1N1 virus at a multiplicity of infection of 0.001 for 90 minutes. After the unadsorbed viruses were washed away, the cell culture was overlaid with 2% agarose to determine the number of the influenza plaques in each well. Data are shown as the mean ± standard error (n = 4).

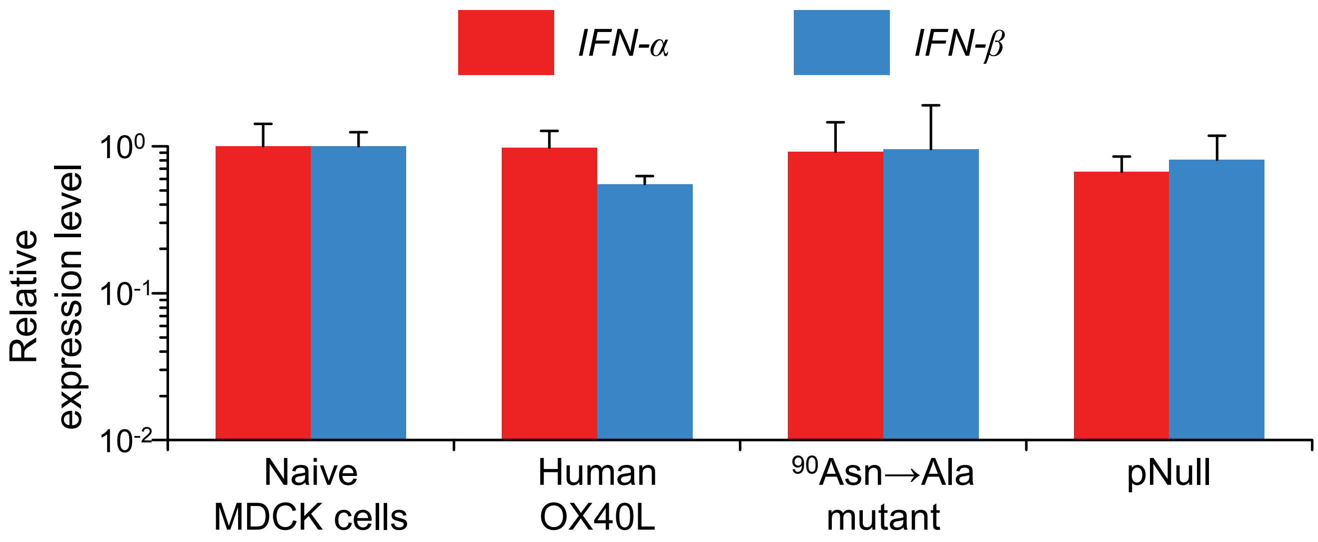

Appendix Figure S8. Type I interferon (*IFN*) genes expression in MDCK cells. The levels of *IFN-α* and *IFN-β* genes expression were analyzed in human OX40L-, ^{90}Asn→Ala mutant-, pNull-transfected MDCK cells by quantitative RT-PCR. They were quantified relative to naive MDCK cells (i.e., no transfection). Data are presented as the mean ± standard error of $n = 3$ per group.

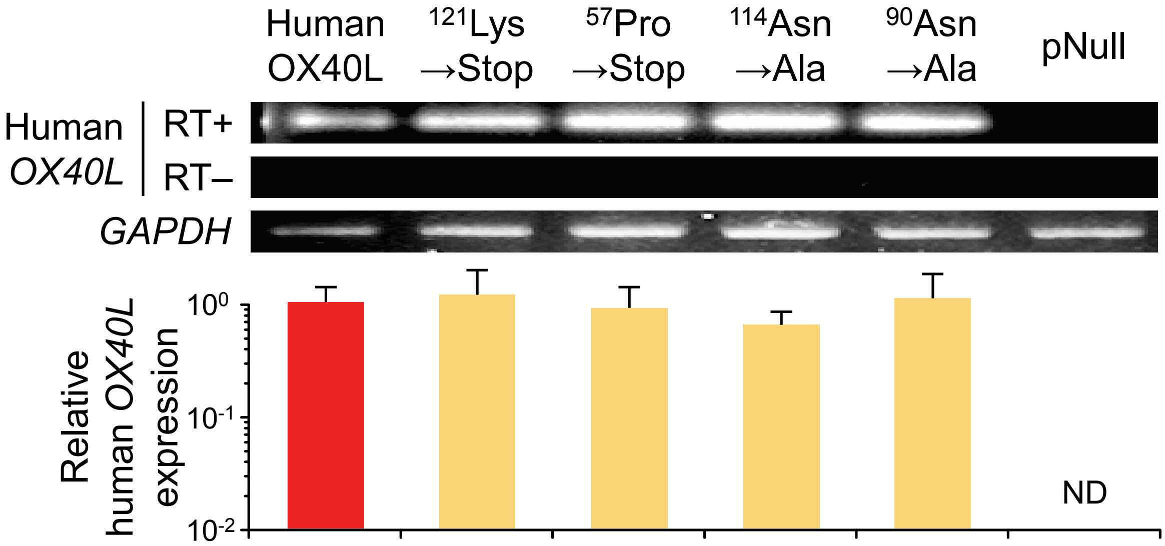

Appendix Figure S9. Wild-type and mutant genes of human OX40L are forcedly expressed at comparable levels. MDCK cells were transfected with mutant human OX40L genes as well as the wild-type one (human OX40L). Controls included pNull-transfected MDCK cells. Twenty-four hours later, the levels of human *OX40L* gene expression were analyzed by semi-quantitative and quantitative RT-PCR with or without reverse transcriptase (RT). The intensity was quantified relative to cells transfected with the wild-type gene of human OX40L. Endogenous canine *GAPDH* mRNA expression was used as a control. Data are shown as the mean ± standard error (n = 3). ND, not detectable.
