# Supplementary material for: OX40 ligand newly expressed on bronchiolar progenitors mediates influenza infection and further exacerbates pneumonia
Source: EMBO Mol Med. 2016 Mar 14;8(4):422–36. doi: 10.15252/emmm.201506154 (PMC4818750; doi:10.15252/emmm.201506154)
Supplement: Supplementary file 2 — Expanded View Figures PDF [file EMMM-8-422-s002.pdf]

Expanded View Figures

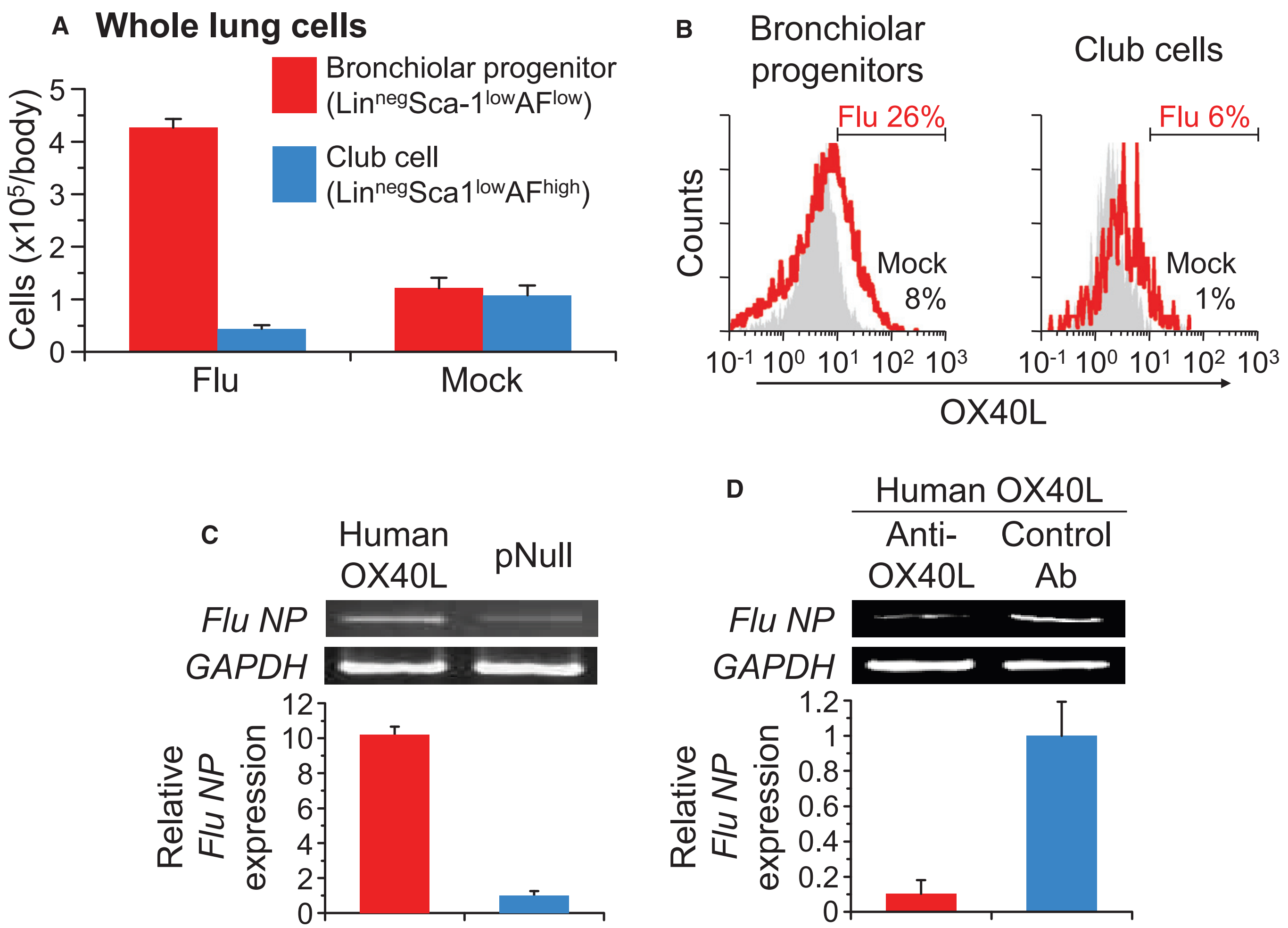

Figure EV1. Influenza A/H3N2 virus infection, which is enhanced by human OX40L expression *in vitro*, increases both the number and the OX40L expression level of bronchiolar progenitors in vivo.

A, B Wild-type mice were intratracheally infected with a lethal dose of influenza A/H3N2 virus (Flu) or saline (Mock), and 7 days later, their lung cells were evaluated for cell counts of bronchiolar progenitors and club cells (A), and OX40L-positive cells in bronchiolar progenitors and club cells (B).

C Human OX40L-transfected MDCK cells were infected *in vitro* with influenza A/H3N2 virus. The levels of influenza virus *NP* gene expression were analyzed by semiquantitative and quantitative RT–PCR 24 h after the infection. The intensity was quantified relative to pNull-transfected cells. Endogenous canine *GAPDH* mRNA expression was used as a control.

D The study was similar to that in panel C, but cells were pretreated with W66 anti-human OX40L antibody for 24 h before the infection. The levels of influenza virus *NP* gene expression were analyzed relative to those in control antibody-pretreated cells.

Data information: Data are presented as the mean ± standard error of n = 4 (A) or n = 3 (C, D) per group.

© 2016 The Authors

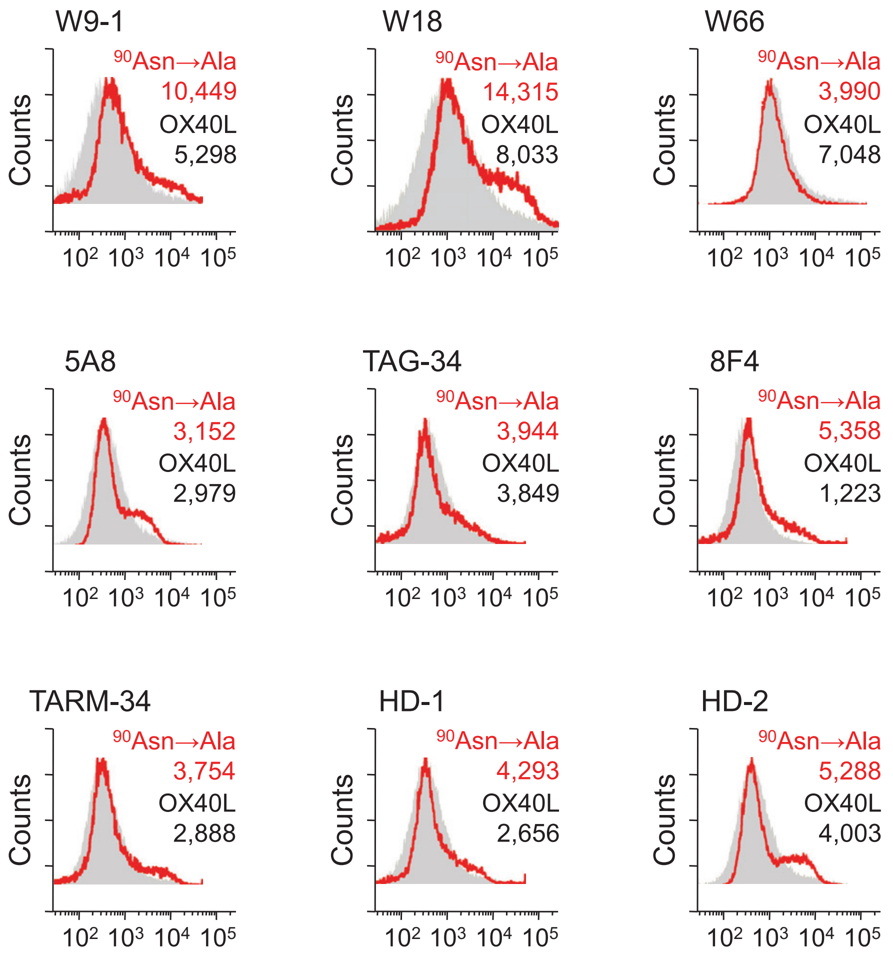

Figure EV2. The mutation of human OX40L at ^{90}asparagine attenuates the binding affinity only for the clone W66 among anti-human OX40L monoclonal antibodies examined.

MDCK cells were transfected with the wild-type human OX40L gene (gray) or the ^{90}asparagine mutant (red, ^{90}Asn→Ala shown in Fig 5D) and were stained with monoclonal antibodies to human OX40L, including clones W9-1, W18, W66, 5A8, TAG-34, 8F4, TARM-34, HD-1, and HD-2. The mean fluorescent intensity was measured by flow cytometry.

© 2016 The Authors
