# Supplementary figures and images for: OX40 ligand newly expressed on bronchiolar progenitors mediates influenza infection and further exacerbates pneumonia
Source: EMBO Mol Med. 2016 Mar 14;8(4):422–36. doi: 10.15252/emmm.201506154 (PMC4818750; doi:10.15252/emmm.201506154)

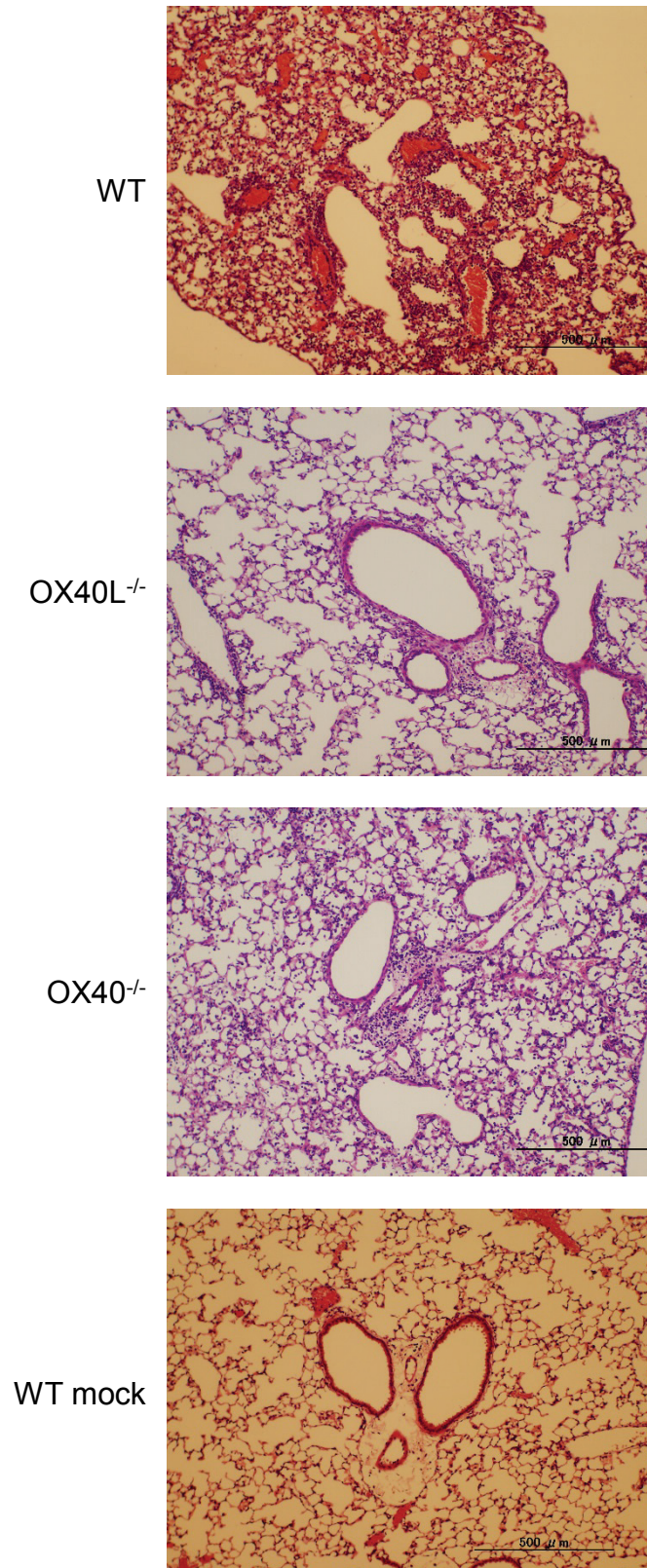

WT → WT

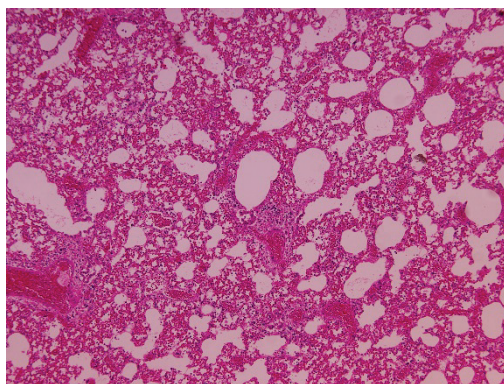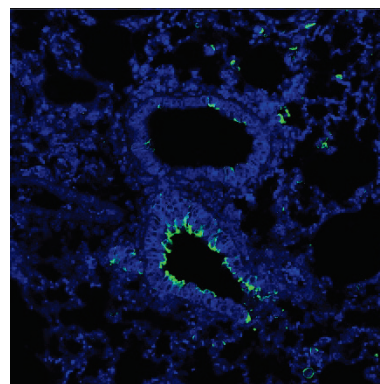

WT → OX40L<sup>-/-</sup>

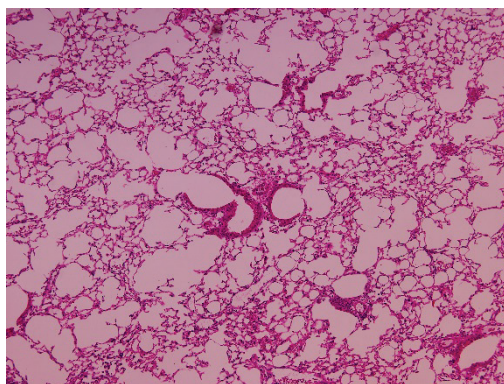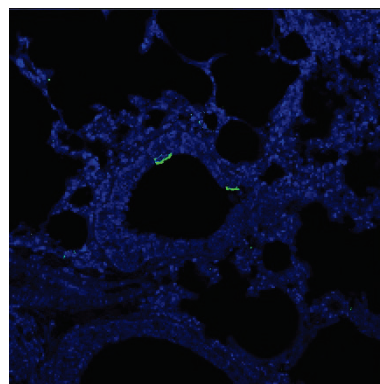

OX40L<sup>-/-</sup> → WT

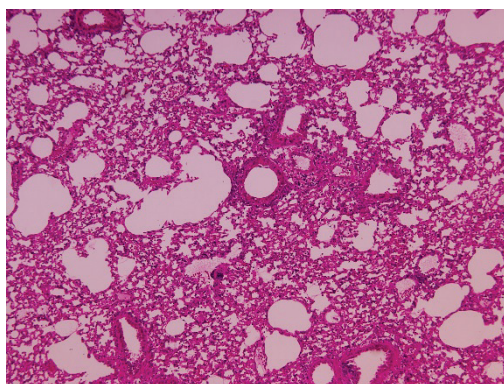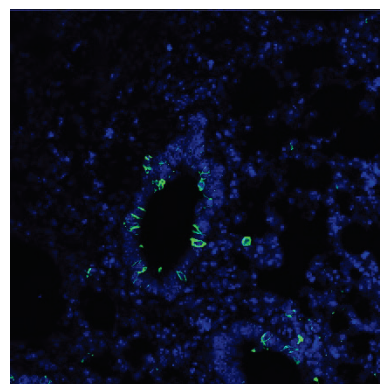

OX40L<sup>-/-</sup> → OX40L<sup>-/-</sup>

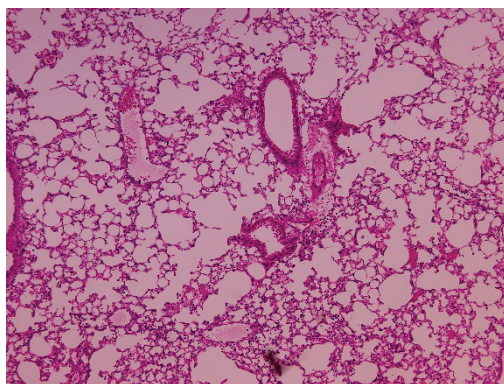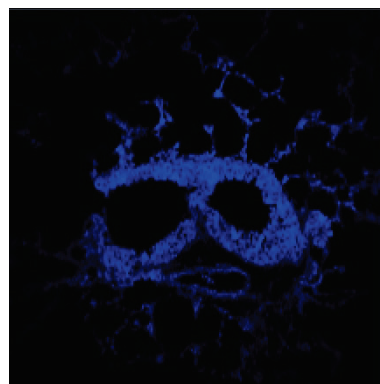

Supplement: Supplementary file 4 — Source Data for Figure 1 [file EMMM-8-422-s003.pdf]

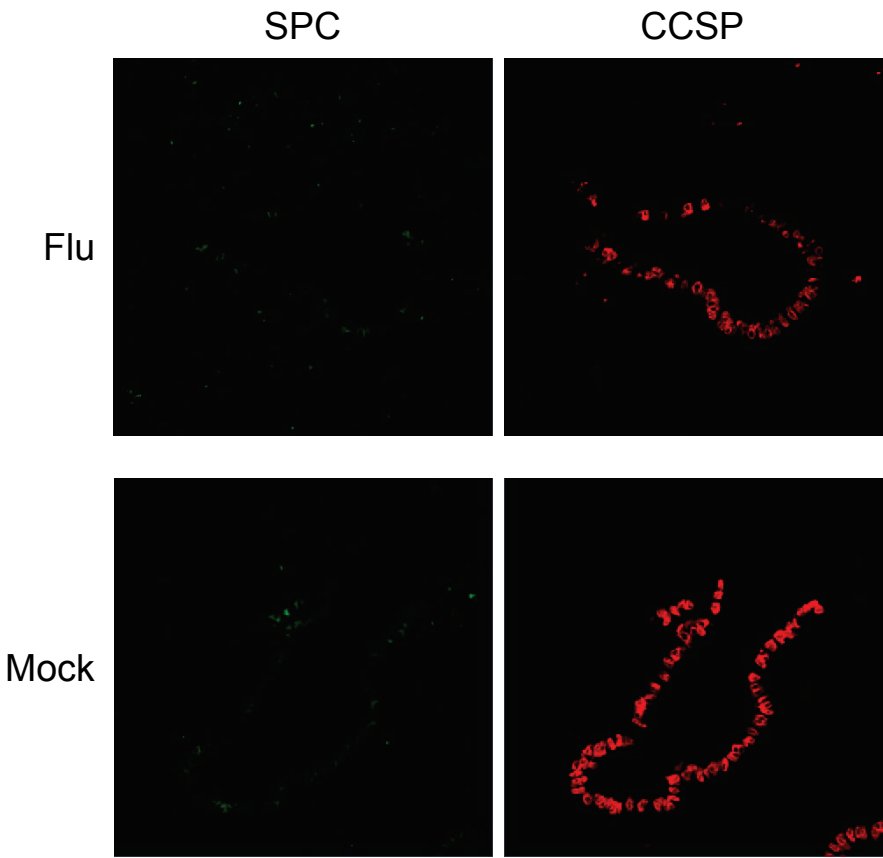

Supplement: Supplementary file 5 — Source Data for Figure 2 [file EMMM-8-422-s004.pdf]

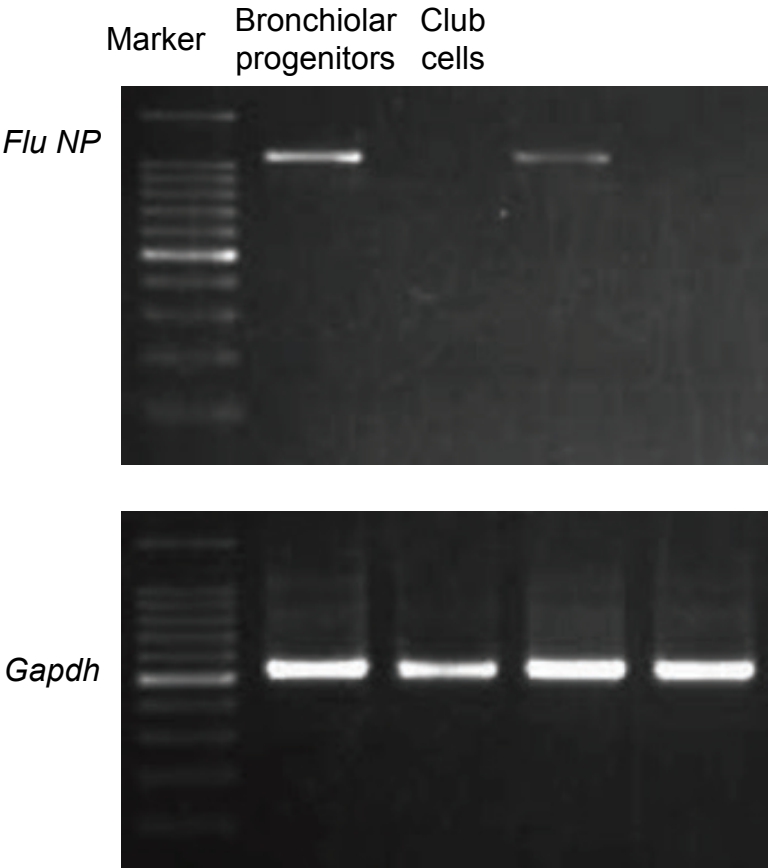

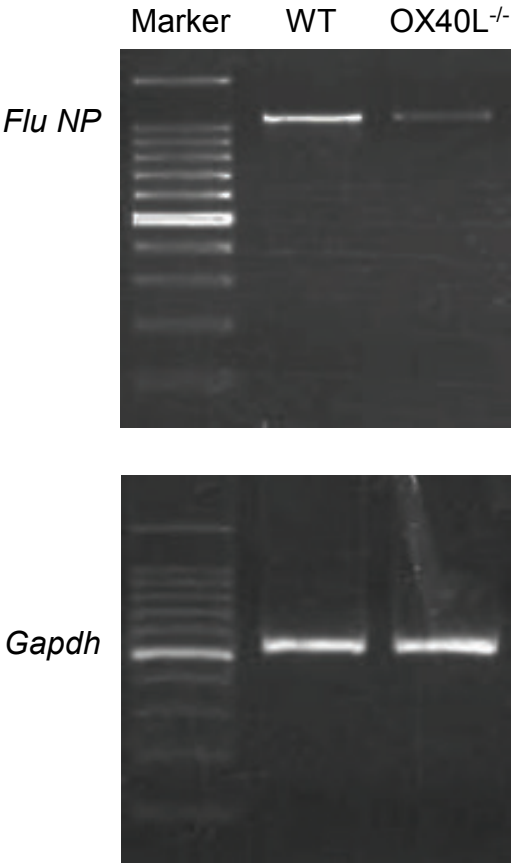

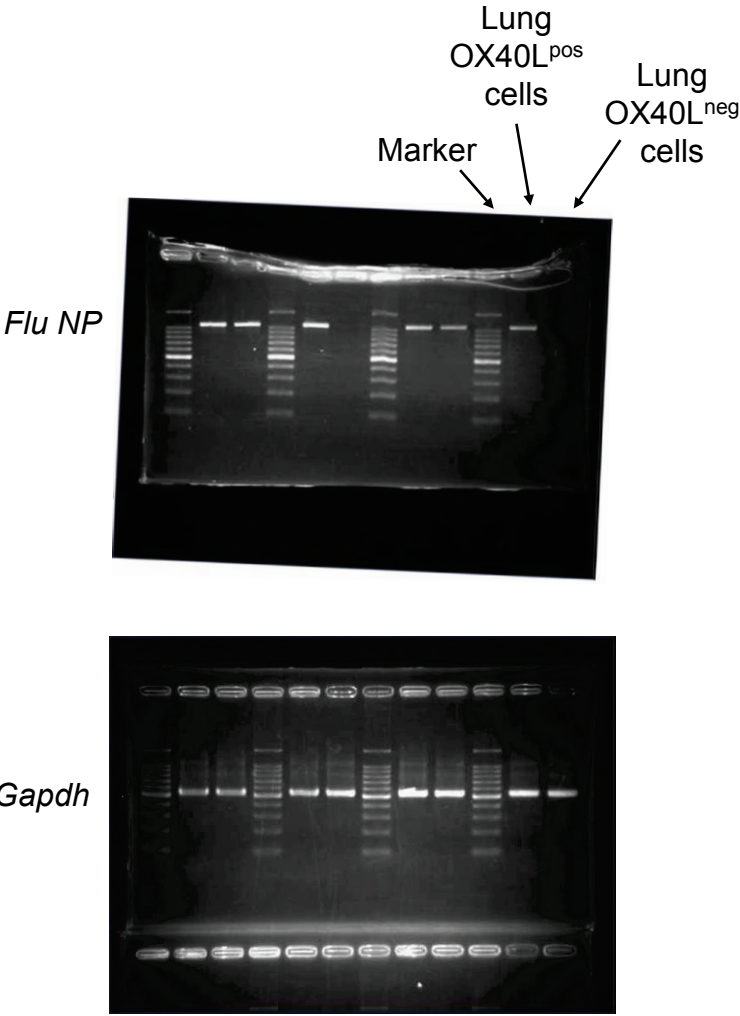

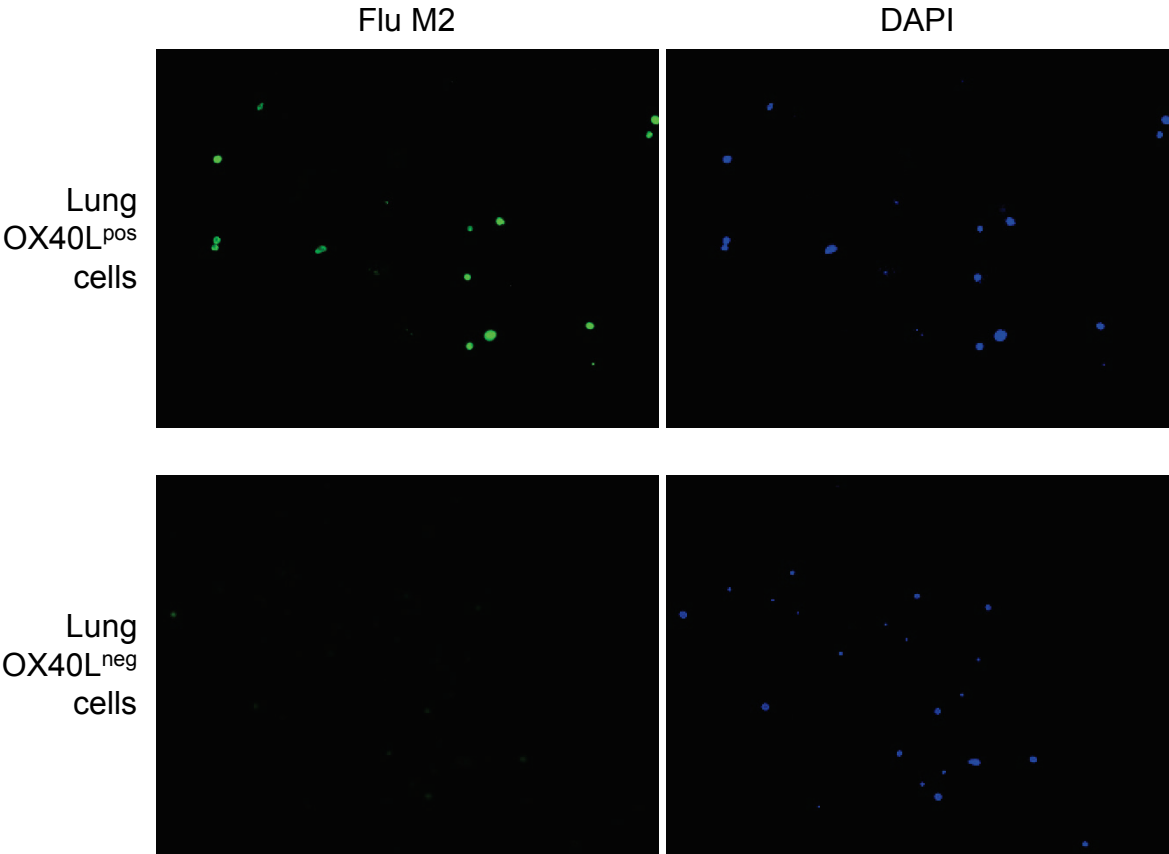

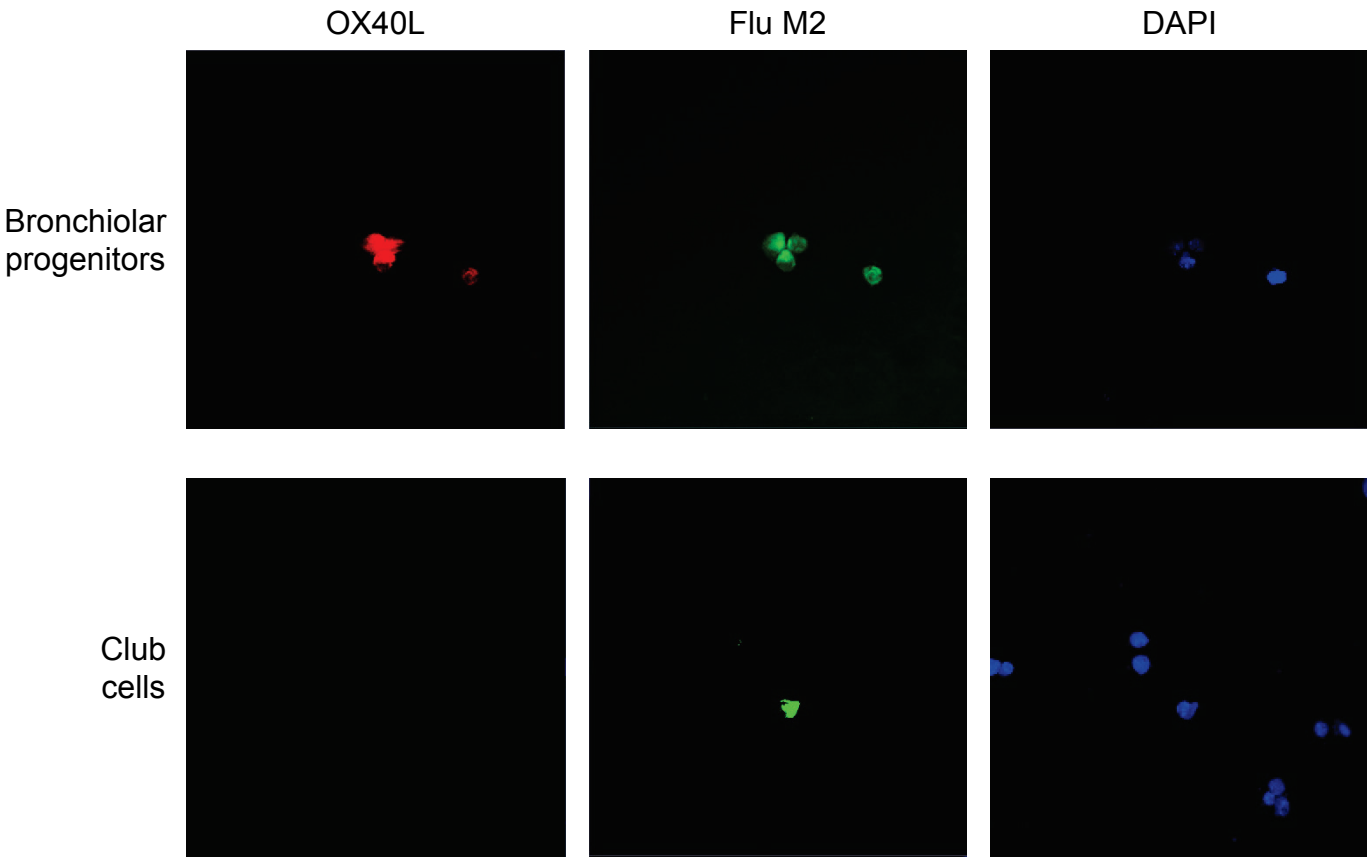

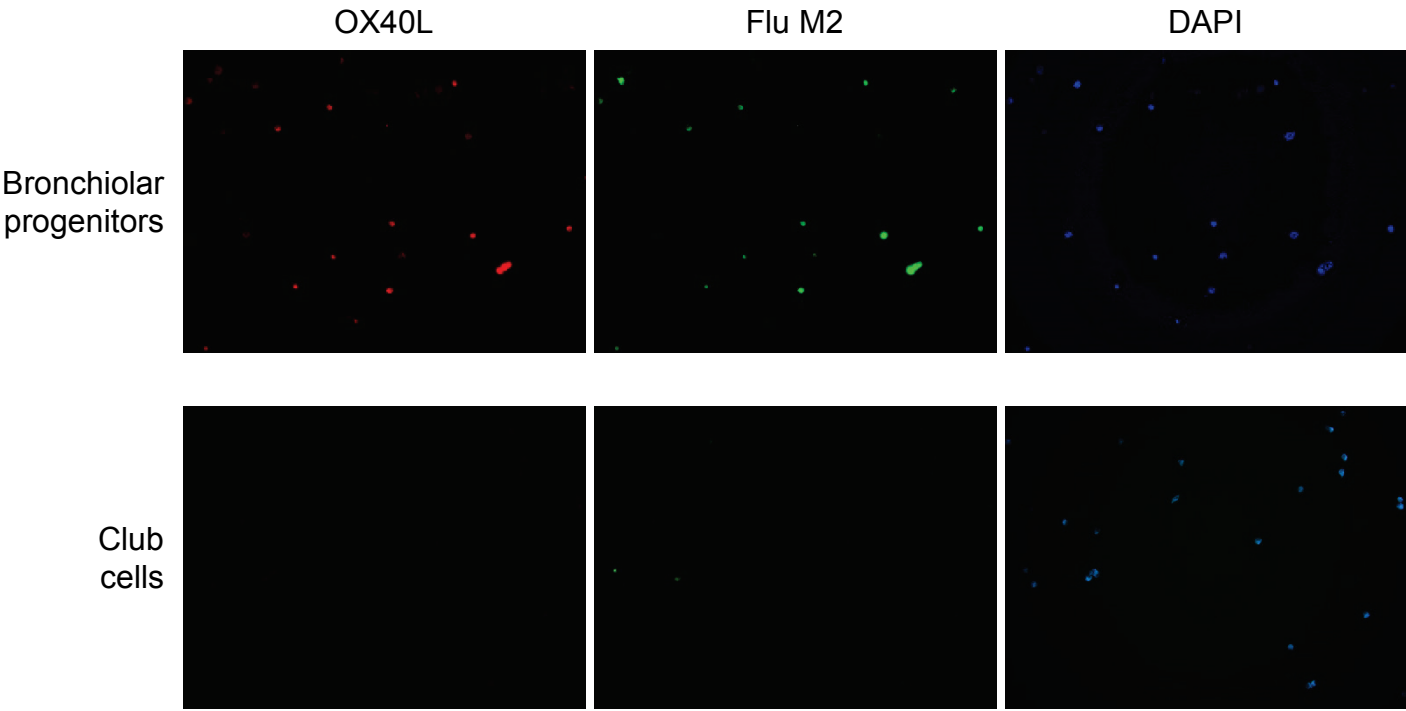

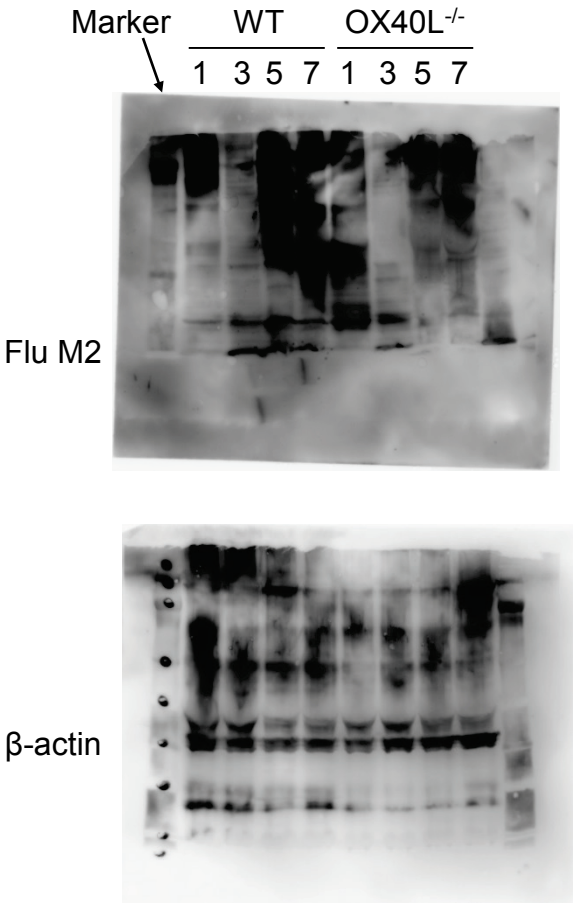

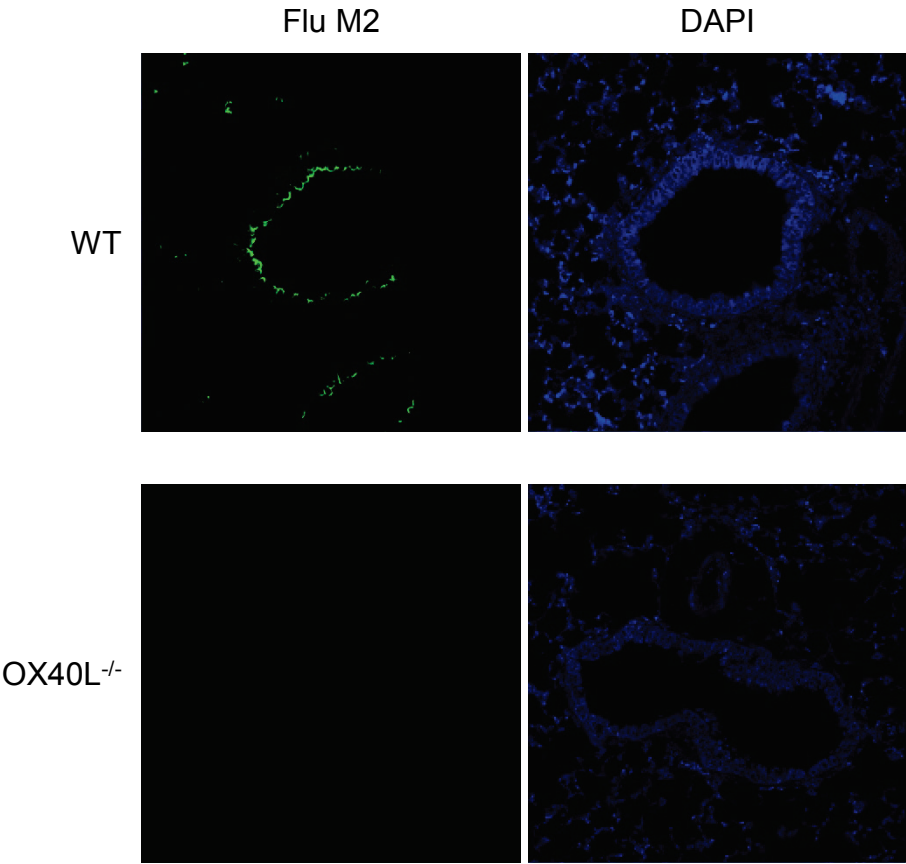

Supplement: Supplementary file 6 — Source Data for Figure 3 [file EMMM-8-422-s005.pdf]

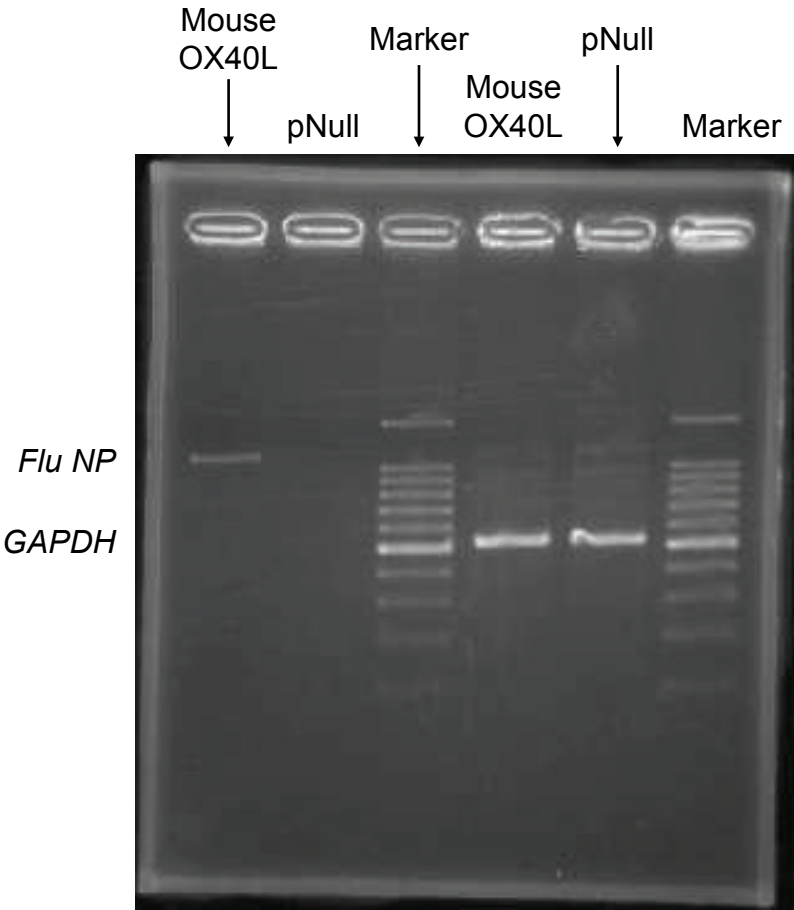

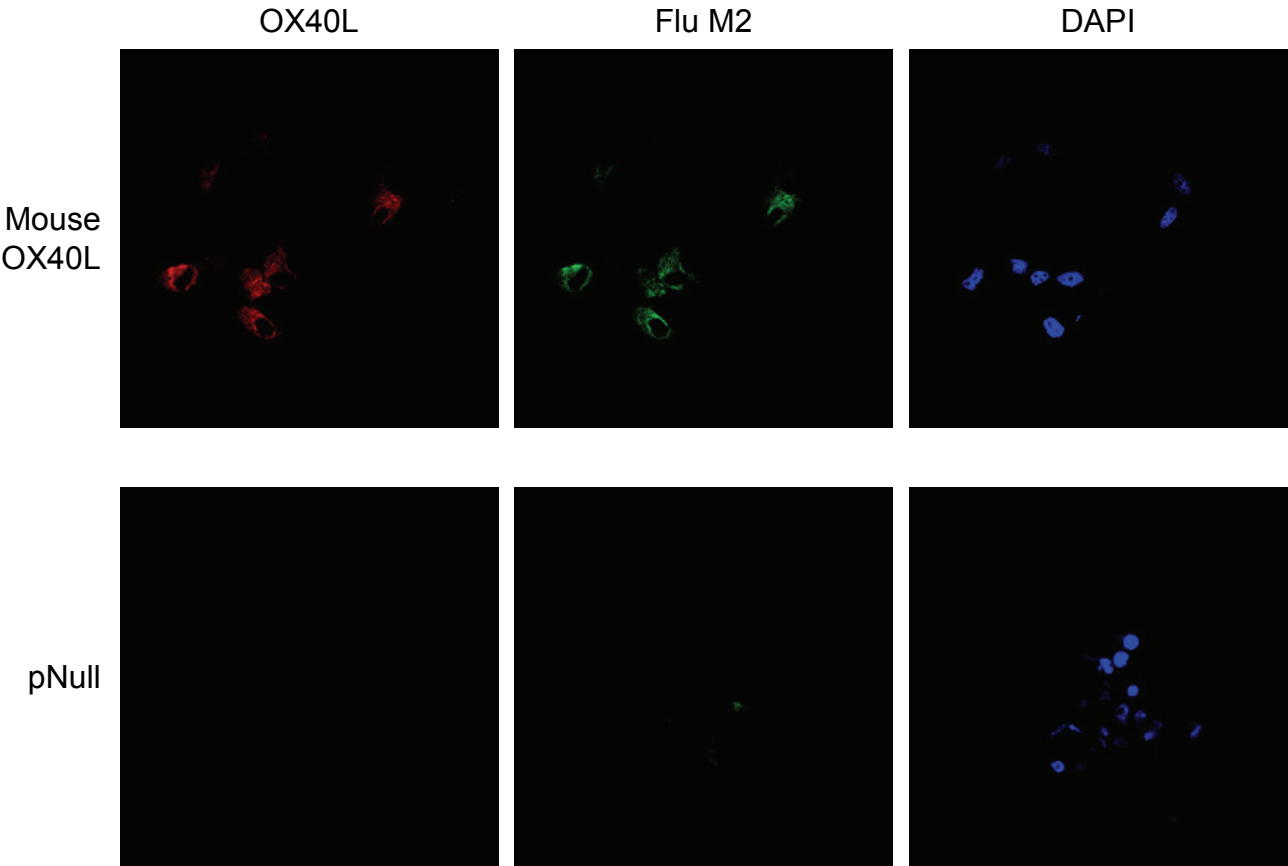

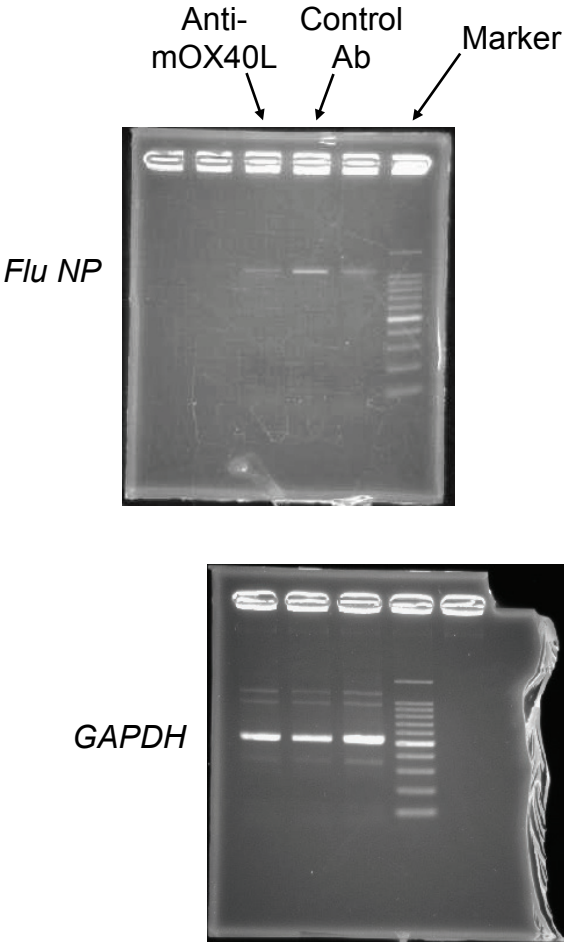

Supplement: Supplementary file 7 — Source Data for Figure 4 [file EMMM-8-422-s006.pdf]

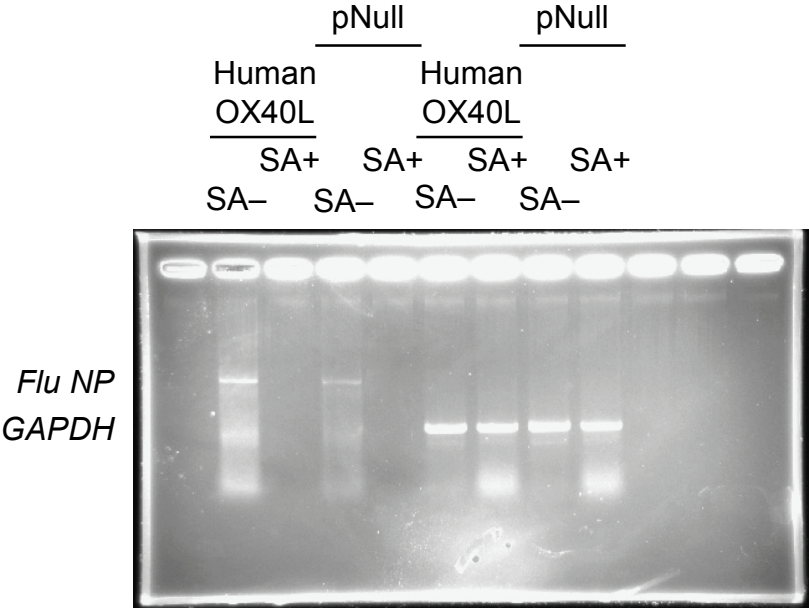

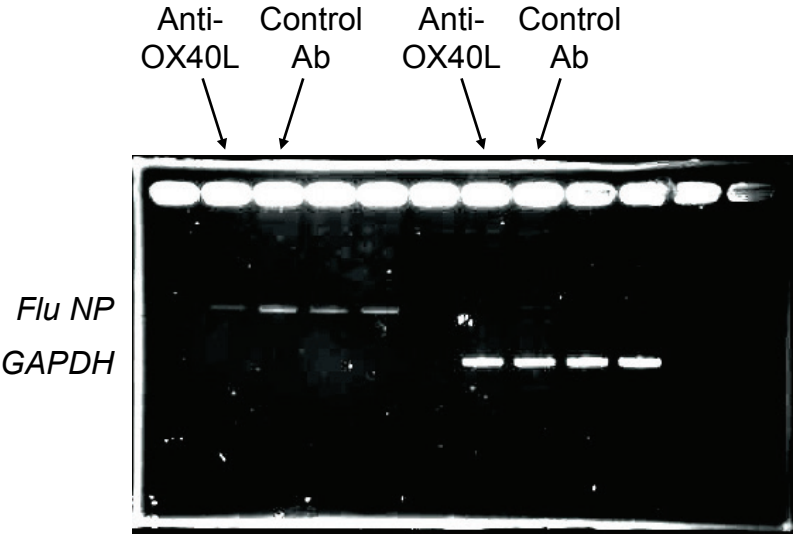

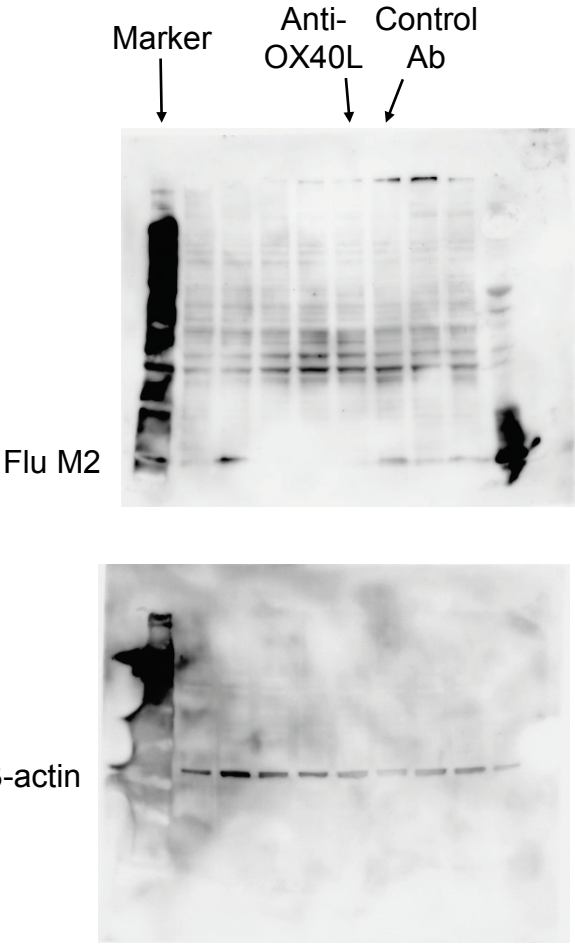

Supplement: Supplementary file 8 — Source Data for Figure 5 [file EMMM-8-422-s007.pdf]
